# Editorial: VDAC Structure and Function: An Up-to-Date View

**DOI:** 10.3389/fphys.2022.871586

**Published:** 2022-04-04

**Authors:** Vito De Pinto, Radhakrishnan Mahalakshmi, Angela Messina

**Affiliations:** ^1^ Department of Biomedical and Biotechnological Sciences, University of Catania, Catania, Italy; ^2^ INBB-Biostructures and Biosystems National Institute, Rome, Italy; ^3^ Molecular Biophysics Laboratory, Indian Institute of Science Education and Research, Bhopal, India; ^4^ Department of Biological, Geological and Environmental Sciences, University of Catania, Catania, Italy

**Keywords:** mitochondria, bioenergetics, outer membrane (OM), voltage dependence, biogenesis, oxidative stress, evolution, cancer biology

The Special Issue “VDAC Structure and Function: an Up-to-Date View” investigated the latest findings obtained on VDAC (Voltage Dependent Anion-selective Channel), i.e., the channel protein allowing the permeability of the outer mitochondrial membrane. The discovery of VDAC in the outer mitochondrial membrane, about 50 years ago (Schein et al., 1976) was a breakthrough either in the evolution studies about the endosymbiotic theory and in the appreciation of organelle’s bioenergetics. Two years ago, we launched this Special Issue about the latest updates in VDAC research in the knowledge that the understanding of the biochemistry of the protein and of the cell physiology linked to its function(s) has not yet reached the desired level. Another aim was to connect and focus the people working on VDAC: it is a fact, indeed, that many groups arrived serendipitously on VDAC and relatively few laboratories in the world are fully concentrated on it. Due to its relative abundance, VDAC was discovered in many diseases and in many cellular districts, but its involvement is not always known in full details. Just to mention an example of a recent high-throughput study that ended up on VDAC, in (Kim et al., 2021) it was found that VDAC3 inhibition has a primary role in TDP-43 pathways during inflammation.

As we wrote in the introduction to the Special Issue, “It is now time to build a unified description of the matter, by joining the contributions of the main laboratories in the world which devoted their efforts into this fascinating protein”.

The excellent contributions have mainly achieved the goal. The introduction is in its historical perspective, which is now well described in the review by Benz, together with those which recently appeared in the literature (De Pinto, 2021; Mannella, 2021): the competition about VDAC discovery and functional elucidation was mainly a matter of few groups. In US three independent laboratories, led by Marco Colombini, Carmen Mannella and Mike Forte, and two in Europe based in Germany and Italy and led by Roland Benz and Vito De Pinto, competed in the discovery and elucidation of biochemical and biomolecular aspects of the VDAC family [see ref. (Colombini and Mannella, 2012) for the history of these achievements]. The knowledge of how research progressed in those years could be of great interest to newcomers to the VDAC field and to young researchers.

Next, as a theme of general interest, the import pathway of VDAC into mitochondria has been described by the group of Rapaport (Moitra and Rapaport) which clarified the role of a β-hairpin motif as an insertion signal, and put forward the model of the lateral gate to mechanistically explain the biogenesis and correct insertion of VDAC in the outer membrane. The work by Court group (Ferens et al.) about the deletion of the 19th strand of the *N. crassa* and its functional consequences upon cell transformation, agrees with those involving human VDAC2 expressed in *S. cerevisiae* (Srivastava and Mahalakshmi, 2020) and does not challenge the results of biogenesis experiments but rather provides suggestive clues about the mechanisms of channel oligomerization.

Another general theme, not frequently dealt with is the genic regulation of VDAC expression. In her work, Zinghirino et al. proposed an overview of theirs and other’s most recent results on the structure of VDAC promoters (Zinghirino et al.). In this paper they compared the three VDAC isoforms regulatory regions, detailing for the first time the presence of isoform-specific cis-acting elements for different transcription factors: they thus paved the way for a deeper understanding of the individual functions of each isoform. In the matter of VDAC isoforms characterization three more contributions described different aspects of this sought information. Magrì et al. reported about the VDAC family in *S. cerevisiae* with a definitive detail, defining the role of each isoform (Di Rosa et al.). Reina and Checchetto reviewed the VDAC3 mammalian isoform, the last to be discovered but target of many recent reports especially dealing with its putative role in the mitochondria redox balance. Also, the contribution by Karachitos et al. tries to explain the reason of the oxidative post-translational modifications of cysteines in VDAC3, in particular. Such redox PTM were subject of a cute Mass Spec study (Pittalà et al., 2021).

The determination of the 3D structure of the pore was a breakthrough in the field of VDAC because allowed to rationalize several hints and information (Hiller et al., 2010). This achievement laid the foundations for VDAC studies related to its involvement in diseases as biomarker or as the target for many new perspective drugs. The work by Shimizu et al. explains the function of a critical residue in VDAC (De Pinto et al., 1993) and connects this study with both the specific function in Ca++ movements in cardiac tissue and the different, antagonistic role of the VDAC isoforms in hearth physio-pathology. The physiological role of VDAC channel in the Ca++ intracellular traffic is an important issue since it is at a cross-road between the storages of the ER and mitochondria (Sander et al., 2021). Another joining point between the physiology and pathologies, where VDAC has a relevant role, was outlined (Heslop et al.; Rostovtseva et al., 2021; Shoshan-Barmatz et al.). The group of Maldonado reviewed the well-established connection between VDAC activity, as the Hexokinase binding receptor, and the Warburg effect, a hallmark of many cancers (Heslop et al.); in this respect the reports about the interaction of mainly disordered peptides, like the C-terminal of tubulin and α-synuclein, with VDAC, shed light on some potentially very important regulatory mechanisms of the pore-activity (Rostovtseva et al., 2021). The influence of a diffuse drug like Metformin on important aspects of mitochondrial dysfunction in energy metabolism and cell death is a clear example of how unexpected might be the VDAC activity (Shoshan-Barmatz et al.).

Last but not least, plant VDACs were described in the review by Pandey, illuminating the similarity/dissimilarities with animal VDACs (Ravi et al.); also, the specific interaction of plant phospholipids with the pore was highlighted and further details emerged from that (Saidani et al., 2021).

This Special Issue was aimed to build a collection of contributions well focused on the state-of-the-art of the structural and functional knowledge of VDAC: we believe it has achieved its goal. It goes without saying that so many answers open up so many new questions. A number of issues, indeed, that make the study of VDAC so topical and fascinating have not yet been resolved: 1) the implementation of voltage dependence in cells and the mechanism by which it occurs; 2) the mobility of the VDAC structure following stimuli or interactions with other proteins; 3) the reported presence of VDAC in other cell membranes and its purpose there. Most likely these topics will be the subject of other bright experiments and other focused Special Issues.
